# Transmission of the PabI family of restriction DNA glycosylase genes: mobility and long-term inheritance

**DOI:** 10.1186/s12864-015-2021-3

**Published:** 2015-10-19

**Authors:** Kenji K. Kojima, Ichizo Kobayashi

**Affiliations:** Department of Computational Biology and Medical Sciences, Graduate School of Frontier Sciences, University of Tokyo, Minato-ku, Tokyo 108–8639 Japan; Institute of Medical Science, University of Tokyo, Minato-ku, Tokyo 108–8639 Japan; Genetic Information Research Institute, Los Altos, CA 94022 USA

**Keywords:** PabI, Restriction endonuclease, Restriction-modification system, DNA glycosylase, DNA methylation, Methyltransferase, *Helicobacter pylori*, *hrgC*

## Abstract

**Background:**

R.PabI is an exceptional restriction enzyme that functions as a DNA glycosylase. The enzyme excises an unmethylated base from its recognition sequence to generate apurinic/apyrimidinic (AP) sites, and also displays AP lyase activity, cleaving the DNA backbone at the AP site to generate the 3’-phospho alpha, beta-unsaturated aldehyde end in addition to the 5’-phosphate end. The resulting ends are difficult to religate with DNA ligase. The enzyme was originally isolated in *Pyrococcus*, a hyperthermophilic archaeon, and additional homologs subsequently identified in the epsilon class of the Gram-negative bacterial phylum Proteobacteria, such as *Helicobacter pylori*.

**Results:**

Systematic analysis of R.PabI homologs and their neighboring genes in sequenced genomes revealed co-occurrence of R.PabI with M.PabI homolog methyltransferase genes. R.PabI and M.PabI homolog genes are occasionally found at corresponding (orthologous) loci in different species, such as *Helicobacter pylori*, *Helicobacter acinonychis* and *Helicobacter cetorum*, indicating long-term maintenance of the gene pair. One R.PabI and M.PabI homolog gene pair is observed immediately after the GMP synthase gene in both *Campylobacter* and *Helicobacter*, representing orthologs beyond genera. The mobility of the PabI family of restriction-modification (RM) system between genomes is evident upon comparison of genomes of sibling strains/species. Analysis of R.PabI and M.PabI homologs in *H. pylori* revealed an insertion of integrative and conjugative elements (ICE), and replacement with a gene of unknown function that may specify a membrane-associated toxin (*hrgC*). In view of the similarity of HrgC with toxins in type I toxin-antitoxin systems, we addressed the biological significance of this substitution. Our data indicate that replacement with *hrgC* occurred in the common ancestor of hspAmerind and hspEAsia. Subsequently, *H. pylori* with and without *hrgC* were intermixed at this locus, leading to complex distribution of *hrgC* in East Asia and the Americas. In Malaysia, *hrgC* was horizontally transferred from hspEAsia to hpAsia2 strains.

**Conclusions:**

The PabI family of RM system behaves as a mobile, selfish genetic element, similar to the other families of Type II RM systems. Our analysis additionally revealed some cases of long-term inheritance. The distribution of the *hrgC* gene replacing the PabI family in the subpopulations of *H. pylori,* hspAmerind, hspEAsia and hpAsia2, corresponds to the two human migration events, one from East Asia to Americas and the other from China to Malaysia.

**Electronic supplementary material:**

The online version of this article (doi:10.1186/s12864-015-2021-3) contains supplementary material, which is available to authorized users.

## Background

Restriction enzymes represent a group of sequence-specific endonucleases that cleave DNA of a specific epigenetic modification state [1], resulting in defense against selfish genetic elements, such as viruses (bacteriophages) and plasmids. A restriction enzyme (R) gene is usually linked with a modification enzyme (M) gene that recognizes the same target sequence and methylates it to prevent cleavage of DNA by the restriction enzyme. Several studies have reported that some restriction-modification (RM) systems are also selfish genetic elements, killing cells that have lost them through chromosome cleavage [2, 3]. Such post-segregational killing is commonly observed with RM systems classified as ‘Type II’. Bioinformatics, molecular evolutionary analyses and laboratory experiments have validated their behavior as mobile genetic elements that insert into and rearrange genomes.

A type II restriction enzyme, R.PabI [4, 5] exhibiting a “half-pipe” fold [6], has been identified as a DNA glycosylase that excises the adenine base from its recognition sequence, GTAC, when not methylated at this site to generate an apurinic/apyrimidinic (AP) site [7, 8]. R.PabI has also been shown to exert AP lyase activity [8]. Until these findings, all restriction enzymes examined had been identified as DNA phosphodiesterases [9]. Despite different ternary structures, all these enzymes cleave the sugar-phosphate backbone of DNA, leaving 5’-phosphate and 3’-OH ends. The AP lyase activity of R.PabI generates a different terminal structure, 5’-phosphate and 3’-phospho alpha, beta-unsaturated aldehyde (3’-PUA) ends [8], which are difficult to re-ligate via DNA ligase. These results led to the reclassification of restriction enzymes into restriction phosphodiesterase and glycosylase subgroups [7, 8].

R.PabI was originally identified from a hyperthermophilic archaeon *Pyrococcus abyssi* during genome comparison with *Pyrococcus horikoshii* [4, 5]. A region including the R.PabI gene and the neighboring methyltransferase gene encoding a DNA methyltransferase, M.PabI, is present in the genome of *P. abyssi*, but absent at the orthologous locus in the genome of *P. horikoshii* [10], which contains an 18 kb sequence instead [5]. Homologs of R.PabI have been detected in various archaea and bacteria, with particular abundance in Epsilonproteobacteria, such as *Helicobacter pylori* [7]. The R.PabI homolog in *H. pylori,* termed R.HpyAXII, is reported to be a GTAC-specific restriction enzyme [11]. The neighboring gene encodes M.HpyAXII, an adenine methyltransferase. These findings have led to the proposal that R.PabI and M.PabI homolog pairs constitute an RM system targeting GTAC tetranucleotides. However, systematic evolutionary analysis of the R.PabI and M.PabI homologs has not been performed to date.

*H. pylori*, a Gram-negative pathogenic bacterium colonizing the stomach, is associated with several gastrointestinal disorders [12]. *H. pylori* is highly divergent in genome sequences within the species [13]. Due to stable transmission from parents to children, the lineages of *H. pylori* maintain traces of human migration. This characteristic was used to reveal human migration to the Americas and Pacific islands [14, 15]. At present, based on the sequences of seven conserved genes, *H. pylori* is classified into several populations (hpAfrica2, hpAfrica1, hpNEAfrica, hpEurope, hpSahul, hpAsia2, and hpEastAsia), each with a distinct geographical distribution [12, 15, 16]. These are further grouped into subpopulations. For example, hpEastAsia is subdivided into hspEAsia, hspMaori, and hspAmerind, consistent with the East Asian origin of Amerind (native American) people [15]. Among these, hspEAsia and hspAmerind are further divided into subgroups [17].

In the current study, systematic examination revealed clear evidence of co-insertion of R.PabI and M.PabI homolog genes. R.PabI and M.PabI homologs in various strains of *H. pylori* and related species, *H. acinonychis* and *H. cetorum*, were orthologous, signifying insertion in the common ancestor. Two examples of cross-genus presence of R.PabI and M.PabI pair at the orthologous locus are also found. Moreover, we showed the geographic and phylogenetic distribution of the *hrgC* gene replacing R.PabI and M.PabI homolog genes in *H. pylori*, and discussed its implications in human migration.

## Results

### Co-occurrence of R.PabI and M.PabI homologs

R.PabI homologs have been identified from various species of archaea and bacteria [7]. Our extensive PSI-BLAST search led to the identification of several homologs from various species. R.PabI homologs were identified for the first time for *Brachyspira* sp., *Mycoplasma primatum*, *Mucispirillum schaedleri*, as well as several species from the genera *Campylobacter* and *Helicobacter* (Additional file 1: Table S1; Additional file 2: Table S2). Analysis of the gene neighborhood of R.PabI homologs showed that almost all R.PabI homologs are next to an adenine methyltransferase gene (Additional file 1: Table S1; Additional file 2: Table S2), as reported for *P. abyssi* and *H. pylori* [5, 10, 11].

**Fig. 1:**
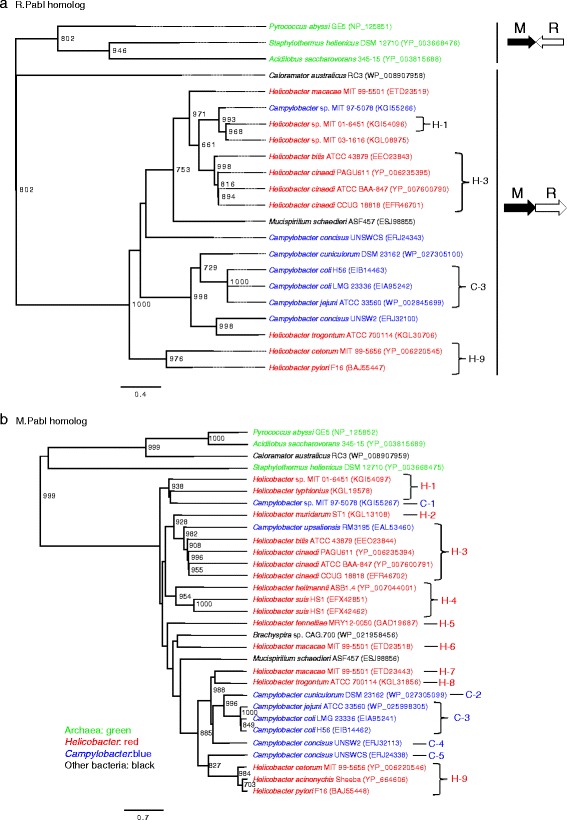
Phylogeny of R.PabI and M.PabI homologs. Trees are rooted at the midpoint. Bootstrap values of 1000 replicates over 50 % are shown at the nodes. Archaeal sequences are colored in green, *Helicobacter* sequences in red and *Campylobacter* sequences in blue. **a** Tree of R.PabI homologs. Four orthologous loci (H-1, H-3, C-3, and H-9) are indicated on the right. The order of R.PabI and M.PabI homolog genes is illustrated on the far right. **b** Tree of M.PabI homologs. Loci are indicated on the right: H-1 to H-9 for *Helicobacter* and C-1 to C-5 for *Campylobacter*. Neighboring genes for each locus are shown in Table 1

Phylogenetic trees were compared based on R.PabI homologs and neighboring methyltransferase genes (Fig. 1). Due to frequent disruption in R.PabI homologs, compared to that in neighboring methyltransferase genes, Fig. 1a includes fewer sequences than Fig. 1b. Hereafter, these methyltransferase genes are designated ‘M.PabI homologs’. BLASTP search with M.PabI as a query led to the identification of several homologous protein sequences that are not associated with R.PabI homologs (data not shown). Using an M.PabI homolog from *H. pylori* as a query, BLASTP hits were mainly proteins associated with R.PabI homologs, and archaeal M.PabI homologs from *Acidilobus* and *Pyrococcus* were hit with e-values of 0.002 and 0.004, respectively. Both archaeal and bacterial M.PabI homologs are gamma-type methyltransferases containing a target recognition domain (TRD) at the C-terminus. Bacterial M.PabI homologs are shorter than their archaeal counterparts due to the shorter C-terminal regions.

Both trees showed two lineages nearly corresponding to archaea and bacteria. Two closely related Epsilonproteobacteria genera, *Campylobacter* and *Helicobacter*, contained almost all the bacterial PabI homologs. It is likely that PabI homologs in *Mucispirillum schaedleri* (Mollicutes) and *Brachyspira* sp. (Spirochaetes) were horizontally transferred from Epsilonproteobacteria. The position of *Caloramator* (Firmicute, Bacteria) was not consistent between the phylogenies of M.PabI and R.PabI homologs, and may represent another lineage distant from archaeal and bacterial lineages or recombination of R.PabI of one subfamily and M.PabI of another (see next paragraph).

The gene order in the PabI family of RM systems corresponded to the phylogeny of M.PabI and R.PabI homologs. The archaeal lineage contained the M and R genes in a tail-to-tail orientation, while the bacterial lineage contained the M gene upstream of the R gene in the same orientation. This finding raises the possibility that archaeal and bacterial M.PabI homologs are not orthologous, and represent two lineages independently paired with R.PabI homologs.

### M.PabI and R.PabI homologs at orthologous sites shared by species

Comparison of *Helicobacter* and *Campylobacter* genomes revealed at least 9 (H-1 to H-9) and 5 (C-1 to C-5) loci for the PabI family of RM systems (Fig. 1 and Table 1). Some of these loci are shared by different species (H-1, H-3, H-4, H-9, C-3) or genera (H-3 and C-1, H-2 and C-4) as detailed below. To confirm the orthology of these cross-species/genera RM systems, we aligned the spacer sequences between the upstream gene and the M gene (Additional file 3: Figure S1), and found that they are very likely orthologous. *Campylobacter upsaliensis* RM 3195 has an M.PabI homolog similar to M.PabI homologs at the H-3 locus in *Helicobacter* (Fig. 1). It was revealed to have been transferred horizontally from *Helicobacter* and thus we included this M.PabI homolog in the H-3 lineage even though it was from *Campylobacter*. The details are described in the next section.

**Table 1:** Loci for the PabI family of RM systems in *Helicobacter* and *Campylobacter*

Locus	Upstream gene	Downstream gene
H-1	Hypothetical protein containing ankyrin repeats	Multidrug transporter MatE/RelE-family toxin
H-2	*Adenylosuccinate lyase*	Hypothetical protein
H-3	*GMP synthase*	Poly A polymerase/Methionyl-tRNA formyltransferase
H-4	Shikimate 5-dehydrogenase	DNA polymerase III subunit delta/Hypothetical protein
H-5	Glutamate synthase large chain	Glutamate synthase small chain
H-6	ABC transporter permease	TetR family transcriptional regulator
H-7	ABC transporter ATP-binding protein	MFS transporter
H-8	Argininosuccinate synthase	Long-chain fatty acid transport protein
H-9	DNA gyrase B	Outer membrane protein 8/Outer membrane protein
C-1	*GMP synthase*	(No sequence information)
C-2	Imidazole glycerol-phosphate dehydratase	Imidazole glycerol phosphate synthase
C-3	Carbamoyl phosphate synthase large subunit	COG0009 Putative translation factor (SUA5)
C-4	*Adenylosuccinate lyase*	Ribonucleotide reductase of class Ia (aerobic), alpha subunit
C-5	RelE family toxin	Hypothetical protein with DUF4279

The closest genes that are not components of RM system are shown. In cases of more than two gene arrangements in the same group, two genes are shown, divided by a slash (/). H-1 to H-9 are in *Helicobacter*, C-1 to C-5 in *Campylobacter*. Orthologous genes seen in different locus groups are italicized (H-2, H-3, C-1 and C-4)

Three closely related M.PabI and R.PabI homologs from two *Campylobacter coli* strains (LMG 23336 and H56) and *C. jejuni* strain ATCC 33560 (C-3 in Fig. 1) were located at the orthologous locus (Additional file 3: Figure S1). However one lineage of *C. coli* (clade I) is reported to have experienced massive introgression from *C. jejuni*, and both *C. coli* strains for C-3 belong to clade I [18]. In fact, no other strains of *C. coli* contained the insertion of the PabI family (data not shown). In view of these results, it is unlikely that insertion of PabI family is an ancestral feature shared by *C. coli* and *C. jejuni*.

**Fig. 2:**
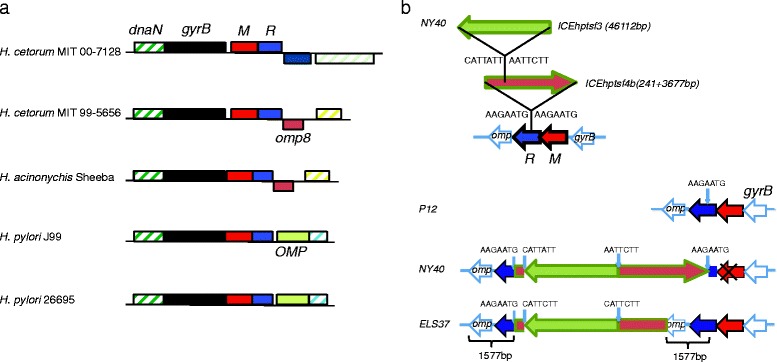
Comparison of structures around the PabI family of RM systems in *H. pylori*, *H. acinonychis* and *H. cetorum*. **a** Genome map comparison around the PabI family. Only the two genes flanking the PabI family at each side are shown. Orthologous genes are presented as boxes of the same color and pattern. Red boxes represent M.PabI homologs and blue boxes are R.PabI homologs. **b** Schematic structure of the ICE insertion within R.PabI homologs in NY40 and ELS37. Each component is not in scale. The upper panel shows nested insertions of two ICEs within the R.PabI homolog gene in NY40. The lower panel shows replacement of the right part of the ICE*hptfs4b* insert in ELS37, compared with NY40

Among the *H. pylori* strains, M.PabI and R.PabI homolog gene pairs were similar. The amino acid identities of M.PabI and R.PabI homologs in J99 (hpWAfrica) and F16 (hspEAsia) were 317/342 (93 %) and 228/251 (91 %), respectively. In *H. pylori*, the M.PabI and R.PabI homolog gene pair was located between DNA gyrase B (*gyrB*) and an outer membrane protein gene represented by HP0506/HP0507 in strain JHP0457 in strain/JHP0457 in strain J99 (Fig. 2a).

The only exception was ELS37 containing two R.PabI homologs, of which one was severely truncated. Their protein sequences were identical in the alignable region. The R.PabI homolog next to M.PabI homolog was intact. Comparison with genomes of the *H. pylori* strains, NY40 and P12, revealed a 1577 bp duplication, coupled with insertion of a ~50 kb mobile genetic element (Fig. 2b). If this insertion was excluded, the PabI family was located at the same site as in other *H. pylori* strains. This mobile genetic element was recently characterized as two integrative and conjugative elements (ICE), designated *ICEhptfs3* and *ICEhptsf4b* [19]. In the genome of NY40, *ICEhptfs4b* was inserted within the R.PabI homolog, duplicating AAGAATG, and *ICEhptfs3* within the *ICEhptfs4B* copy, with AAGAATG target site duplication in the opposite orientation. Duplicated sequences were mutated in NY40 to AAGAATT and AATAATG. Here, the R.PabI homolog gene was split into two segments, and the M.PabI homolog gene disrupted by mutations. In the ELS37 genome, the 1654 bp right terminal region of *ICEhptsf4b* was replaced by a duplicated 1577 bp segment containing part of the R.PabI homolog gene and part of an outer membrane protein gene. Thus, this duplication possibly occurred via a gene conversion-like mechanism with incoming DNA of intact R.PabI homolog as the template. The right junction appeared to result in homologous recombination and the left junction in non-homologous recombination. This recombination event resulted in a 1577 bp duplication sandwiching the ~50 kb ICE insertion.

In the genomes of *H. acinonychis* and *H. cetorum*, two species closely related to *H. pylori*, the M.PabI and R.PabI homolog gene pair was located downstream of *gyrB* (Fig. 2a). The gene downstream of the PabI homolog gene pair was outer membrane protein 8 (*omp8*), which is absent in *H. pylori*. The intergenic sequence between *gyrB* and the M.PabI homolog was shorter than 10 bp and appeared conserved, except for *H. cetorum* MIT 99–5656, which contained a sequence of unknown origin between the two genes (Additional file 3: Figure S1). Among these species, *H. pylori* and *H. acinonychis* are reported to be close, while *H. cetorum* is more divergent [20]. Data from our phylogenetic analysis of M.PabI homologs were consistent with this relationship (Fig. 1). The most parsimonious explanation is that the PabI family was integrated between *gyrB* and *omp8* in the common ancestor of *H. pylori*, *H. acinonychis* and *H. cetorum*, and the downstream region rearranged. The results collectively suggest that the PabI family has been maintained for a long time in these species. Linkage of RM systems with genome rearrangement junctions has been reported [21].

### M.PabI and R.PabI homologs at orthologous sites shared by genera

H-3 in the genus *Helicobacter* and C-1 in *Campylobacter* occur downstream of a homologous gene for GMP synthase (Table 1). Although GMP synthase in C-1 corresponds to only a C-terminal fragment of the complete protein, the two GMP synthase proteins are 88 % identical in the alignable region. The junctions between the genes for GMP synthase and M.PabI homolog in H-3 and C-1 resembled each other to a significant extent (Additional file 3: Figure S1). A sequence almost identical to the junction sequence in H-3 was found in *C. upsaliensis* RM3195, followed by a gene for the M.PabI homolog (Fig. 1b, Additional file 3: Figure S1). This M.PabI homolog (EAL53460) displayed 89 % amino acid sequence identity to that from *H. cinaedi* PAGU611. This M.PabI homolog is encoded near a contig end, and the partner R.PabI homolog is not on the contig. Instead, we identified a fragmented R.PabI homolog (EAL52983) coded at the end of another contig (Table S1). This R.PabI homolog is ~96 % identical to those in *H. cinaedi* and ~93 % to that in *H. bilis*. Therefore it is very likely that EAL53460 and EAL52983 are an M.PabI and R.PabI homolog pair.

Other *C. upsaliensis* strains, JV21 and DSM 5365, lacked the M.PabI and R.PabI homolog pair, but their sequences for GMP synthase were more similar to those of RM3195 and some *Helicobacter* species than those of other *Campylobacter* species. It indicates that the GMP synthase gene was horizontally transferred from *Helicobacter* to the common ancestor of *C. upsaliensis*. In the three strains of *C. upsaliensis*, the GMP synthase gene is followed by a non-homologous gene, indicating genome rearrangement downstream of the GMP synthase gene. A single event of horizontal transfer of the GMP synthase gene as well as the PabI family and subsequence recombination leading to the loss of the PabI family in some strains is more likely, compared with two independent horizontal transfer events (the GMP synthase gene and the PabI family) to the same genomic locus. Thus, we conclude that the H-3 lineage is horizontally transferred from *Helicobacter* to *C. upsaliensis* with the flanking GMP synthase gene.

The origin of insertion at C-1 in *Campylobacter* sp. MIT 97-5078 is unknown at present. One possibility is that the PabI family was present in the common ancestor of *Campylobacter* and *Helicobacter*, and has been lost in many species/strains. We cannot eliminate another horizontal transfer between *Helicobacter* and *Campylobacter*, but even so, the transfer must be very old, considering their sequence diversity. Long-term maintenance of the PabI family at the orthologous locus is the most likely scenario.

Similarly, two adenylosuccinate lyase proteins encoded upstream of C-4 and H-2 displayed 73 % identity. The genome of *H. muridarum* have 6 M (pseudo)genes and 4 R (pseudo)genes between two non-RM genes there (Additional file 4: Figure S2). Here, recurrent acquisition of different RM systems has obscured the relationship between C-4 and H-2 (Additional file 3: Figure S1), but we cannot exclude the orthology of these two loci.

### Gene neighborhood of the PabI family of RM systems

In H-5 and C-2, PabI family is located in a putative operon whose genes are tightly linked in function and encoded on the same strand (Table 1). Glutamate synthase large chain and small chain are components of the same enzyme for glutamate metabolism. Both imidazole glycerol phosphate dehydratase and imidazole glycerol phosphate synthase play a role in histidine metabolism. In these cases, the RM system is coded on the same strand as the neighboring genes. This type of operon insertion has been reported for other RM systems and operons [22], and may impose operon maintenance and expression through post-segregational killing.

The PabI family is occasionally linked with other RM or toxin-antitoxin systems. The genes encoding defense system components, such as RM, toxin-antitoxin, and CRISPR-Cas systems typically cluster in “defense islands” [23]. The PabI family in *H. cinaedi* PAGU611 is flanked by an RM system. The PabI family in *C. cuniculorum* DSM 23162 replaced a restriction enzyme gene (see next section for details). The extreme case is observed in *H. muridarum* (Additional file 4: Figure S2). Between two non-RM genes, there are 6 M (pseudo)genes and 4 R (pseudo)genes. The PabI family of H-1 and C-5 is flanked by genes for the RelE toxin family. These two toxin genes show very weak sequence similarity to each other (data not shown), indicating they represent independent association of RM system and RelE toxin genes.

### Insertion of PabI family of RM system

**Fig. 3:**
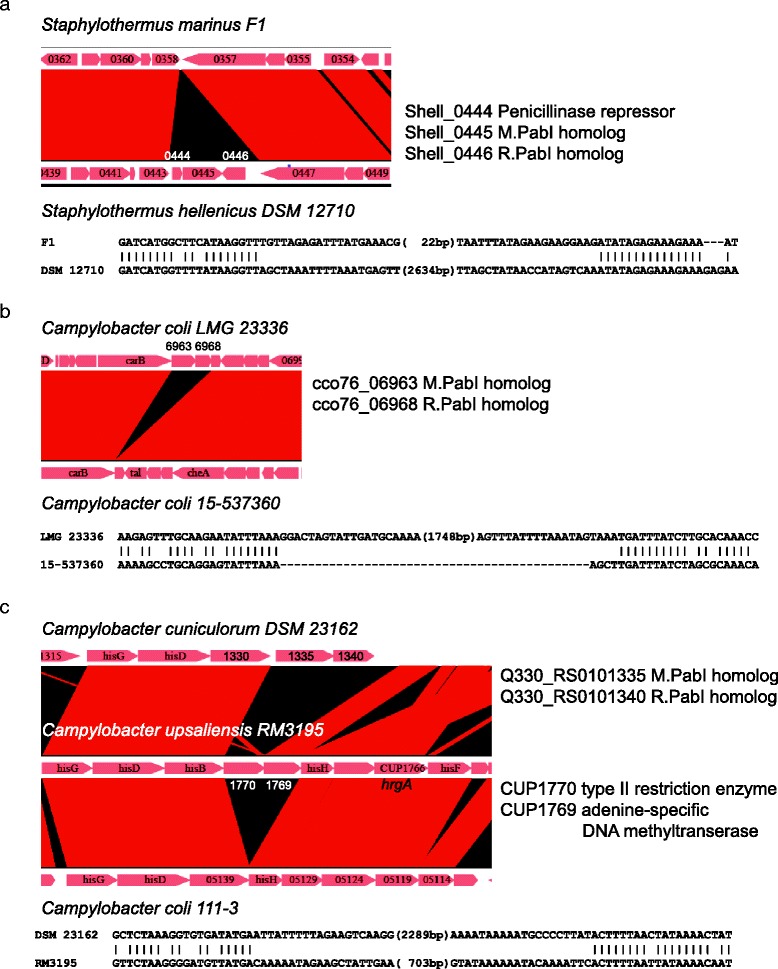
Integration of the PabI family of RM systems. Two genome maps are compared. Red boxed arrows indicate genes with coding directions. Gene numbers or names are presented in or near the arrows. Regions of sequence similarity between loci are indicated by red bands. Junction sequences are shown below the genome map comparison. Genes within the insertions with their annotations are shown on the right. **a** Genome map comparison of *Staphylothermus marinus* F1 and *S. hellenicus* DSM 12710 around the PabI family in the *S. hellenicus* genome. **b** Genome map comparison of *Campylobacter coli* LMG 23336 and *C. coli* 15–537360 around the PabI family in the LMG 23336 genome. **c** Genome map comparison of *Campylobacter cuniculorum* DSM 23162, *C. upsaliensis* RM3195 and *C. coli* 111–3 around the PabI family in the DSM 23162 genome

Genome sequence comparison among closely related species/strains revealed the mobility of the PabI family (Fig. 3). Comparison of *Staphylothermus hellenicus* DSM 12710 and *S. marinus* F1 revealed insertion into *S. hellenicus* of three ORFs: R.PabI and M.PabI homologs and part of a penicillinase repressor (Fig. 3a). This PabI RM system replaced a 62 bp DNA segment.

Comparison of *Campylobacter coli* LMG 23336 (locus C-3) and 15–537360 strains disclosed a sequence insertion only including two genes for R.PabI and M.PabI homologs (Fig. 3b). This RM system replaced the 4 bp sequence, AGCT. All *C. coli* strains available at the NCBI BLAST website other than LMG 23336 and H56 displayed a conserved structure identical to 15-537360. TAAA tetranucleotides were observed at the insertion boundary, although it is unclear whether they represent target site duplications.

Comparison of *Campylobacter cuniculorum* DSM23162 (locus C-2) and *C. upsaliensis* RM3195 revealed replacement of a putative Type II RM system R gene with a PabI family (Fig. 3c). The (presumably cognate) M gene neighboring the replaced R gene and flanking *hrgA* gene were disrupted in the DSM23162 genome. The *hrgA* gene was previously reported to replace a restriction enzyme gene in *H. pylori* [24], but the *hrgA* gene seen here does not replace an RM system. The putative Type II RM system itself was possibly inserted in the ancestral locus of these two genomes, since *Campylobacter coli* 111–3 lacks the Type II RM system. According to REBASE (http://tools.neb.com/blast/), this putative RM system recognizes 5’GANTC.

### Identification of two members of the PabI family of RM system in *H. macacae* MIT 99-5501

We identified two PabI family members from *H. macacae* MIT 99-5501 (Table 1 and Fig. 1, loci H-6 and H-7). One R.PabI homolog (annotated as two proteins, ETD23441 and ETD23442) was disrupted, but the other R.PabI homolog (ETD23519) and both M.PabI homologs (ETD23443 and ETD23518) appeared intact. Phylogenetic analysis (Fig. 1) indicated that these two members of the PabI family were independently acquired by *H. macacae*. One M.PabI homolog (ETD23443), associated with a disrupted R.PabI homolog, was most closely related to that from *Helicobacter trogontum* (KGL31856), and the other (ETD23518) neighboring an intact R.PabI homolog (ETD23519) related to that from *Brachyspira* (WP_021958456).

### Shared rearrangement in the PabI family of RM system in hspEAsia and hspAmerind

**Fig. 4:**
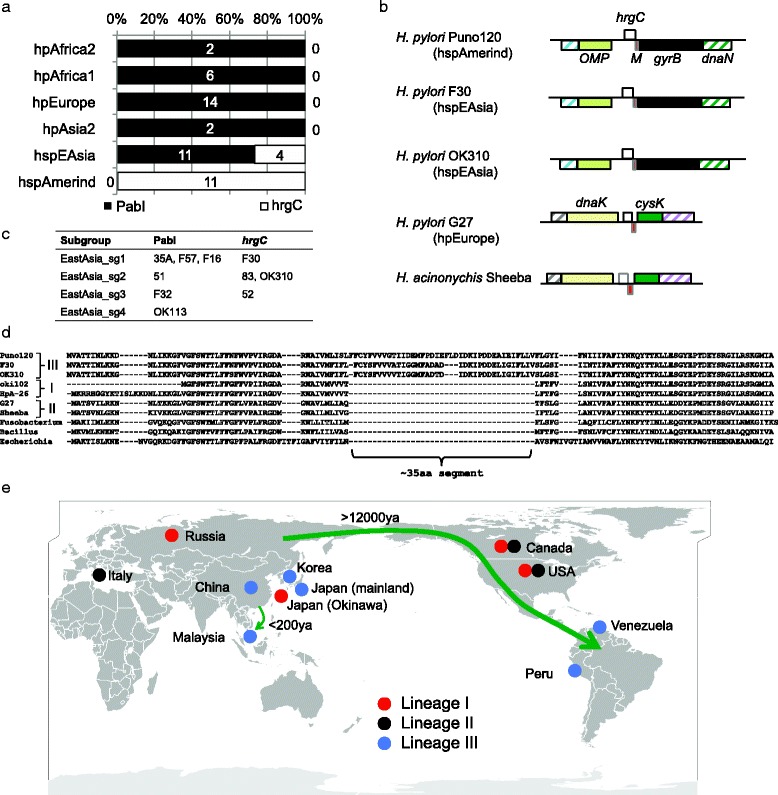
Replacement of the PabI family of RM system with *hrgC*. **a** Distribution of the PabI homolog vs. *hrgC* gene in each population of *H. pylori*. Only strains that have been completely sequenced and deposited in RefSeq were counted. Analyzed strains are documented in Additional file 2: Table S2. **b** Genome map comparison around *hrgC* genes. Orthologous genes are shown as boxes with the same color and pattern. White boxes represent *hrgC* genes. **c** Distribution of the PabI homolog vs. *hrgC* gene in East Asia subgroups of *H. pylori*. Only strains reported in Yahara et al. [17] are analyzed. **d** Alignment of HrgC proteins. The C-terminal 15 residues of the *Escherichia* HrgC protein are omitted. Roman numbers right of the *H. pylori* and *H. acinonychis* strain names indicate the *hrgC* lineages shown in Fig. 5. Accession numbers are as follows. Puno120, AEN15115; F30, BAJ56916; OK310, BAM98251; oki102, AHN34853; HpA-14, WP_000301367; G27, ACI26869; Sheeba, YP_665190; Fusobacterium, YP_008019852; Bacillus, EJS64418; Escherichia, AIT14382. **e** Geographic distribution of *hrgC* gene. Three lineages of *hrgC* are colored differently. Green arrows indicate two human migration routes

Some R.PabI homolog genes in *H. pylori* are disrupted due to frameshift, nonsense mutations, and/or deletions (data not shown). All hspAmerind strains sequenced completely to date contain a large deletion within the PabI family (Fig. 4a). The same deletion is observed in some hspEAsia strains, such as 52 (Korea), 83, F30 (Fukui, Japan) and OK310 (Okinawa, Japan). Sequence comparisons revealed that these deletions are caused by replacement with a DNA segment containing one gene, annotated *hrgC* (Fig. 4b). While this substitution was previously reported [11], its geographical distribution remains to be established. Among the complete genomes for *H. pylori*, only hspAmerind and hspEAsia strains contained *hrgC* (Fig. 4a), placed in an opposite orientation to the M.PabI and R.PabI homologs. hspEAsia is subdivided into 4 subgroups in Yahara et al. [17], and no subgroup show uniform presence/absence of *hrgC* (Fig. 4c).

The *hrgC* gene encodes a small protein with a length of <100 residues (Fig. 4d). Although the function of *hrgC* gene has not been confirmed, its homologs have been identified in various bacteria, including *Escherichia coli* and *Bacillus cereus*, indicating horizontal transfer of the gene. Secondary structure prediction with jpred3 (http://www.compbio.dundee.ac.uk/www-jpred/) [25] indicated that the HrgC protein contains one transmembrane helix. This result is consistent with the annotation of some HrgC homologs as inner membrane proteins. These features are found in toxins of type I toxin-antitoxin systems [26], leading to the speculation that HrgC is a toxin of a toxin-antitoxin system (see Discussion).

The 5’-terminal 78 bp M.PabI and 3’-terminal 88 bp R.PabI homolog genes were observed at the locus in these *hrgC*-encoding strains, indicating that the PabI family is replaced by the *hrgC* gene and not vice versa (Additional File 5: Figure S3).

*hrgC* was also located between *dnaK* and *cysK* in the genome of *Helicobacter acinonychis* Sheeba as well as several *H. pylori* strains, such as G27 (Fig. 4b, d). This finding is in keeping with the report that *H. acinonychis* and *H. pylori* strain NSH57 (a derivative of G27) contain the *hrgC* gene at a locus distinct from the “*pabI*” locus [11]. The locus is occupied by a gene that encodes a protein similar to the N-terminal region of McrB in most *H. pylori* strains. McrB is a DNA binding subunit of Type IV (methylation-specific) restriction endonuclease that acts in concert with McrC [22, 27]. Notably, the 166 bp 5’ fragment of the M.PabI gene was associated with the *hrgC* gene at the “*mcrB*” locus (Additional file 5: Figure S3). Compared with HrgC proteins encoded at the “*pabI*” locus, those encoded at the “*mcrB*” locus lacked a ~35 aa segment (Fig. 4d, G27 and Sheeba). This difference may be due to an insertion in the lineage at the “*pabI*” locus, since HrgC proteins encoded within the genomes of other species do not contain a sequence corresponding to this 35 aa segment. Thus, insertion of 35 aa segment is likely a derived feature.

The TBLASTN search with HrgC protein as a query against the Whole genome shotgun sequence (wgs) database led to the identification of several more *H. pylori* strains containing the *hrgC* gene. These sequences were encoded at either the “*pabI*” or “*mcrB*” locus (data not shown). Interestingly, HrgC proteins encoded at the “*pabI*” locus in some strains lacked the ~35 aa segment (Fig. 4d, oki102 and Hp A-26), including four strains (oki102, oki112, oki422 and oki898) from Okinawa, Japan, three from North America (R056a, R018c and Hp A-26), 173/00 from Portugal, and A45 from Russia. In summary, HrgC proteins with the ~35 aa segment have been identified from Japan, Korea, China, Malaysia, and South America (Fig. 4e).

### HrgC phylogeny

**Fig. 5:**
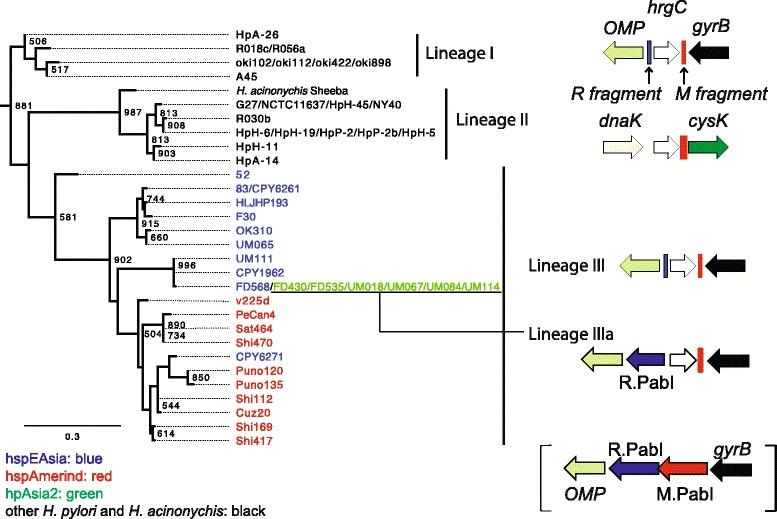
Phylogeny of HrgC proteins from *H. pylori* and *H. acinonychis*. Seven bacterial HrgC sequences (YP_004074033, YP_008019852, WP_025697443, KEJ93376, KGG55171, EJS64418, and AIT14382) were used as outgroups to determine the root. Bootstrap values of 1000 replicates over 50 % are shown at the nodes. Strain names belonging to hspEAsia are colored in blue, hspAmerind in red and hpAsia2 in green. The gene map near *hrgC* is illustrated on the right. Lineage IIIa corresponding to the hpAsia2 strains shows a chimeric structure of the locus containing *hrgC* (Lineage III) and that containing the PabI family (in brackets)

To trace the origin of replacement of the PabI family of the RM system with the *hrgC* gene, we performed phylogenetic analysis based on the HrgC protein sequences (Fig. 5). As expected, all HrgC proteins from *H. pylori* and *H. acinonychis* clustered together. The most distant lineage included HrgC proteins encoded at the “*pabI*” locus and lacking the ~35 aa segment (lineage I in Figs. 4 and 5). To confirm their distant phylogenetic positions, we generated another phylogenetic tree based on sequences excluding the ~35 aa segment, with the same topology (data not shown). The phylogenetic position of the HrgC proteins encoded at the “*mcrB*” locus (lineage II) was unexpected considering their locus different from those of the other lineages (Lineages I and III), both of which are located at the same “*pabI*” locus, but this position is not well statistically supported.

HrgC protein sequences from hpAsia2 strains were identical to those from some hspEAsia strains. Among the Malaysian strains, three (UM065, UM111, FD568) from the Malaysian Chinese population belonged to hspEAsia [28, 29]. Five strains (UM018, UM067, UM114, FD430, FD535) from Malaysian Indians and one strain (UM084) from Malays belonged to hpAsia2 (hpSouthIndia) [28, 29]. Two (FD535 and UM084) encoded 208 of 251 aa of the R.PabI homolog besides HrgC (lineage IIIa in Fig. 5). The remaining four hpAsia2 strains (UM018, UM067, UM114, and FD430) displayed a 124 bp deletion within the R.PabI homolog coding region. These findings collectively suggest horizontal transfer of the *hrgC* gene from hspEAsia to hpAsia2.

## Discussion

### The PabI family of RM systems displays characteristics similar to other RM systems

The PabI family is unique, encoding a sequence-specific DNA glycosylase/AP lyase as the restriction enzyme [7, 8]. All other reported RM systems examined to date encode a certain type of DNA phosphodiesterase [9]. Despite fundamental differences in catalytic activity, the PabI family shares many features with other RM systems. Genes for PabI family of restriction glycosylase and adenine methyltransferase genes are tightly associated (Additional file 1: Table S1; Additional file 2: Table S2). Phylogenetic analyses support the theory that that they move together as a unit (Fig. 1). Genome comparisons of closely related strains revealed insertion of the glycosylase and methyltransferase gene pair (Fig. 3). These features are shared with other RM systems.

### Cross-species and cross-genus distribution of orthologous PabI family of RM systems

Orthologous insertions of the closely related PabI family across species are observed in *Helicobacter* and *Campylobacter* species (H-1, H-3, H-4, H-9 and C-3 in Fig. 1, Table 1 and Additional file 3: Figure S1). C-3 represents recent horizontal transfer between species, while H-3 and H-9 possibly indicate insertion in the common ancestor.

Both H-3 in the genus *Helicobacter* and C-1 in *Campylobacter* are located downstream of an orthologous gene for GMP synthase. We cannot exclude the possibility that this reflects a preference for the insertion site by the PabI family, but in that case, the underlying molecular mechanism or possible biological significance (such as selective advantage) remains to be established. Recent horizontal transfer between different genera cannot explain the sequence divergence between H-3 and C-1 in *Campylobacter* sp. MIT 97–5078. The similarity of the C-1 locus in a strain of *C. upsaliensis* (RM3195) shows a clear case of horizontal transfer from *Helicobacter* to *Campylobacter* (Additional file 3: Figure S1). It is likely that the presence of the PabI family downstream of the GMP synthase gene represents an ancient insertion that has been maintained in some strains of these two genera via vertical transmission, possibly coupled with horizontal transfer. Another possibility is an ancient event of horizontal transfer between genera. In our knowledge, there is no report showing long-term maintenance of RM systems leading to their cross-genus distribution. This is the first report of such maintenance. We do not consider it is related to the exceptional catalytic activity of R.PabI homolog, and are convinced that many more cases of long-term maintenance of RM systems be found in the near future.

### Replacement of the PabI family of RM system with an *hrgC* gene provides clues on human migration history

In some *H. pylori* strains, the PabI family was replaced by an *hrgC*-coding DNA segment. Phylogenetic analysis revealed three *hrgC* lineages in *Helicobacter* (Fig. 5, lineages I–III). The history of lineage III is easy to reconstruct (Fig. 4e). A strain of *H. pylori* acquired the *hrgC* gene that replaced part of the PabI family in East Asia. One of its descendant lineages moved to the Americas, forming the hspAmerind lineage. The uniform presence of *hrgC* in the hspAmerind lineage is consistent with the isolation of Amerind people in the Americas. In contrast, the other descendant lineage was mixed with other hpEastAsia strains in East Asia, leading to sporadic distribution of the *hrgC* gene in hspEAsia. Finally, Chinese people moved to Malaysia and the *hrgC* gene in the hspEAsia strain was transferred to the hpAsia2 strain. This transfer left a fragment of R.PabI homolog, indicating that the replacement is initiated at the M.PabI homolog side (Fig. 5). A previous analysis of *H. pylori* in Southeast Asia revealed that Malaysian hspEAsia strains migrated with Chinese people from Guangzhou and Hong Kong, China within the last 200 years [30]. Thus the transfer of *hrgC* from hspEAsia to hpAsia2 was a quite recent event. It agrees with the completely identical protein sequences of HrgC among the hpAsia2 strains. The transfer of DNA segments occurs generally between family members [31]. Thus the presence of *hrgC* gene in hpAsia2 strains indicates the intermarriage of Chinese immigrant and either Malay or Indian immigrant. The information for such a single event may be lost in the genome-wide study.

In lineage II, the position of *H. acinonychis* Sheeba is the most divergent. The clustering of all *H. pylori* strains is reasonable. The origination of the lineage II *hrgC* gene remains an open question. Lineages I and III contain the *hrgC* gene at the “*pabI*” locus. The simplest explanation is that *hrgC* gene moved from “*pabI*” to the “*mcrB*” locus in the common ancestor of lineage II. Notably, lineage II has the 166 bp fragment of M.PabI homolog, while both I and III contain only the 78 bp fragment of M.PabI homolog (Additional file 5: Figure S3). Thus, the possibility that the PabI family may have once been inserted at the “*mcrB*” locus and was replaced by *hrgC* in lineage II cannot be discounted.

Lineage I lacks the ~35 aa segment at the “*pabI*” locus. Thus, if replacement of PabI family with *hrgC* occurred only once, the lineage would retain the original structure. This lineage was obtained from Japan, Russia and North America (Fig. 4e), and their genomes are related to those of hpEurope strains based on the ribosomal protein sequences (NCBI Genome Groups, http://www.ncbi.nlm.nih.gov/genome/?term=helicobacter%20pylori). The geographic distribution of lineage I raises the possibility that this sequence was derived from a population in Siberia, and shares the origin with lineage III, which was originated in East Asia.

HrgC was similar to toxins of type I toxin-antitoxin systems in size, with a secondary structure suggestive of a transmembrane helix [26] and apparent mobility between genomes [23]. The toxin-antitoxin and RM systems are analogous from many viewpoints, including attack on host bacteria and gene regulation pathways sometimes involving antisense RNA [32, 33]. The genes encoding defense system components, such as RM, toxin-antitoxin, and CRISPR-Cas systems, typically cluster in islands called “defense islands” [23]. Our findings may indicate stepwise evolution of coupling and co-transfer of RM and toxin-antitoxin systems. More specifically, the promoter for M.PabI homolog may provide an antisense RNA (antitoxin) suppressing the expression of HrgC, a putative toxin. Inhibition of this promoter would lead to attack on the host through post-segregational killing in the PabI family, as well as the composite of *hrgC* (hypothetical toxin gene) and M.PabI homolog fragment. This may represent takeover of an RM system by a type I toxin. This is just a speculation at present, not supported by any experimental data, but it is worth mentioning to encourage further study. Linkage of the PabI family of the RM system with a toxin gene (Table 1) may have similar significance.

## Conclusions

R.PabI is an exceptional restriction enzyme that acts as a sequence-specific DNA glycosylase. Despite their unique characteristics, R.PabI homologs show similar ways of existence to restriction DNA phosphodiesterases, including co-occurrence and co-mobilization with a methyltransferase (M.PabI homolog) gene, and linkage with the genome rearrangement junctions. These findings are consistent with the theory that the PabI family of RM system is a typical mobile selfish genetic element.

Despite the obvious mobility between genomes, the PabI family of RM system is occasionally observed at the same locus of different strains, species or even genera. This may be attributed to ancient insertion of these RM systems that are maintained via vertical transmission, probably coupled with occasional horizontal transfer via homologous recombination.

The PabI family of RM system is occasionally replaced by an *hrgC* gene-containing DNA segment in *H. pylori* strains. This substitution possibly occurred in the common ancestor of hspEAsia and hspAmerind. The hspAmerind strains retain this structure owing to geographic isolation, while only a small proportion of hspEAsia strains retain the *hrgC* gene, indicating that their admixture with strains diverged earlier than the substitution event. In Malaysia, *hrgC* was horizontally transferred from hspEAsia to hpAsia2 strains. The genetic admixture of *H. pylori* strains would have occurred in an individual or a population, revealing traces of human migration history.

## Methods

### Detection of R.PabI homolog and gene neighborhood analysis

R.PabI homologs were searched using iterative PSI-BLAST [34] with R.PabI as a query until no more proteins were hit over the threshold e-value 0.005. Once all R.PabI homologs were obtained, the genomic sequences were downloaded from the NCBI website (http://www.ncbi.nlm.nih.gov). At least two annotated genes at each side of R.PabI were obtained manually, and analyzed for methyltransferases.

The orthology of neighboring genes was initially examined based on their annotation, and protein sequences aligned to confirm their sequence identity.

### Genome comparison among closely related species/strains

TBLASTN search was performed with protein sequences encoded each side of R.PabI homolog genes against genome sequences available on the NCBI website. When both genes were hit to the same genomic sequence of a strain, the relevant sequences were downloaded and analyzed manually. Sequence similarity was visualized with the aid of GenomeMatcher [35]. The ends of sequence identity were determined based on BLASTN results.

### Phylogenetic analysis

Genes of R.PabI and M.PabI homologs, including frameshift mutations or large deletions, were excluded from phylogenetic analysis. HrgC protein sequences hit in the BLASTP search with the HrgC protein of *H. pylori* strain F30 as a query were downloaded from the NCBI website. Multiple alignments of full-length R.PabI homologs, M.PabI homologs and HrgC proteins were generated using MAFFT with the linsi option [36]. Phylogenetic analysis was performed on the PhyML server with the LG or WAG model and 1000 bootstrap replicates [37]. The two models resulted in very similar topology in all cases, and therefore only the LG trees were used. A multiple alignment excluding the ~35 aa segment of HrgC proteins was also generated with aLRT (alternative likelihood ratio test) values. Trees were drawn using FigTree v1.4.2 (http://tree.bio.ed.ac.uk/software/figtree/).

### Comparison of orthologous loci among H. pylori strains

Orthologous loci in *H. pylori* were determined based on the Microbial Genome Database for Comparative Analysis (MBGD) website (http://mbgd.genome.ad.jp/) [38], and examined manually.

### Availability of supporting data

Datasets supporting the results are included within the article and additional files.

## Additional files

Additional file 1: Table S1. R.PabI and M.PabI homologs outside *H. pylori.* Accession numbers for fragmented proteins are shown in parentheses. (PDF 83 kb) Additional file 2: Table S2. M.PabI and R.PabI homologs in the RefSeq genomes of *H. pylori*, *H. acinonychis* and *H. cetorum*. Sheeba, *H. acinonychis*; MIT 99–5656, *H. cetorum.* All other strains are *H. pylori*. Strains belonging to hspEAsia are colored in blue and those belonging to hspAmerind in red. In cases where more than two genome sequences are reported for one strain, only one genome is shown. (PDF 86 kb) Additional file 3: Figure S1. Intergenic sequences between orthologous M.PabI homologs and upstream genes. Start codons for the M.PabI homologs are in bold and stop codons for upstream genes are shaded. Under the nucleotide sequences, encoded protein sequences are shown. (PDF 76 kb) Additional file 4: Figure S2. Accumulation of R and M genes around the PabI family of RM system in *H. muridarum*. The arrows represent genes. Gene numbers are shown above the arrows. The R and M gene pairs are indicated by brackets. Target sequences are presented below the RM pairs or M genes. Gene 3185 is a partial duplication of gene 3170. The sequences of genes 3210 and 3175 showed no significant sequence similarities. (PDF 878 kb) Additional file 5: Figure S3. Sequence comparisons of M.PabI and R.PabI homolog fragments. Sequences of *H. acinonychis* Sheeba and *H. pylori* G27 are in the “*mcrB*” locus, while other sequences are in the “*pabI*” locus. The sequences that do not correspond to M.PabI or R.PabI homologs are colored in either red or blue. Nucleotides distinct from the M.PabI or R.PabI homolog sequence are shaded in the alignable region. The start codon (TTG) for M.PabI homolog and stop codon (TAA) for R.PabI homolog are in bold. (PDF 56 kb)

## Abbreviations

RM system: Restriction-modification system
AP lyase: Apurinic/apyrimidinic lyase
3’-PUA: 3’-phospho alpha, beta-unsaturated aldehyde
ICE: Integrative and conjugative element
