# Supplementary material for: Transmission of the PabI family of restriction DNA glycosylase genes: mobility and long-term inheritance
Source: BMC Genomics. 2015 Oct 19;16:817. doi: 10.1186/s12864-015-2021-3 (PMC4615327; doi:10.1186/s12864-015-2021-3)
Supplement: Additional file 1: Table S1. — R.PabI and M.PabI homologs outside H. pylori. Accession numbers for fragmented proteins are shown in parentheses. (PDF 83 kb) [file 12864_2015_2021_MOESM1_ESM.pdf]

Table S1. R.PabI and M.PabI homologs found outside of *H. pylori.*

Species	Strain	R.PabI homolog	M.PabI homolog
Pyrococcus abyssi	GE5	NP_125851	NP_125852
Acidilobus saccharovorans	345-15	YP_003815688	YP_003815689
Staphylothermus hellenicus	DSM 12710	YP_003668476	YP_003668475
Caloramator australicus	RC3	WP_008907958	WP_008907959
Brachyspira sp.	CAG:700	(WP_021958455)	WP_021958456
Mycoplasma primatum	ATCC 25948	(WP_029513686/WP_029513685)	(WP_029513687)
Campylobacter coli	LMG 23336	EIA95242	EIA95241
Campylobacter coli	H56	EIB14463	EIB14462
Campylobacter jejuni	ATCC 33560	WP_002845699	WP_025998305
Campylobacter concisus	UNSW2	ERJ32100	ERJ32113
Campylobacter concisus	UNSWCS	ERJ24343	ERJ24338
Campylobacter sp.	MIT 97-5078	KGI55266	KGI55267
Campylobacter cuniculorum	DSM 23162	WP_027305100	WP_027305099
Campylobacter upsaliensis	RM3195	(EAL52983)	EAL53460
Mucispirillum schaedleri	ASF457	ESJ98855	ESJ98856
Helicobacter acinonychis	Sheeba	(YP_664607/YP_664608)	YP_664606
Helicobacter cetorum	MIT 99-5656	YP_006220545	YP_006220546
Helicobacter bilis	ATCC 43879	EEO23843	EEO23844
Helicobacter cinaedi	PAGU611	YP_006235395	YP_006235394
Helicobacter cinaedi	ATCC BAA-847	YP_007600790	YP_007600791
Helicobacter cinaedi	CCUG 18818	EFR46701	EFR46702
Helicobacter fennelliae	MRY12-0050	(GAD19686)	GAD19687
Helicobacter heilmannii	ASB1.4	(YP_007044002)	YP_007044001
Helicobacter macacae	MIT 99-5501	(ETD23442/ETD23441)	ETD23443
Helicobacter macacae	MIT 99-5501	ETD23519	ETD23518
Helicobacter muridarum	ST1	(Disrupted)	KGL13108
Helicobacter suis	HS1	(Disrupted)	EFX42851
Helicobacter suis	HS5	(Disrupted)	EFX42462
Helicobacter trogontum	ATCC 700114	KGL30706	KGL31856

Helicobacter typhlonius		(Disrupted)	KGL19578
Helicobacter sp.	MIT 01-6451	KGI54096	KGI54097
Helicobacter sp.	MIT 03-1616	KGL08975	Not annotated

Accession numbers for fragmented proteins are shown in parentheses.
