# Supplementary material for: Transmission of the PabI family of restriction DNA glycosylase genes: mobility and long-term inheritance
Source: BMC Genomics. 2015 Oct 19;16:817. doi: 10.1186/s12864-015-2021-3 (PMC4615327; doi:10.1186/s12864-015-2021-3)
Supplement: Additional file 2: Table S2. — M.PabI and R.PabI homologs in the RefSeq genomes of H. pylori, H. acinonychis and H. cetorum. Sheeba, H. acinonychis; MIT 99–5656, H. cetorum. All other strains are H. pylori. Strains belonging to hspEAsia are colored in blue and those belonging to hspAmerind in red. In cases where more than two genome sequences are reported for one strain, only one genome is shown. (PDF 86 kb) [file 12864_2015_2021_MOESM2_ESM.pdf]

Table S2. M.PabI and R.PabI homologs in the RefSeq genomes of *H. pylori*, *H. acinonychis* and *H. cetorum*.

Strain[1]	RefSeq	R.PabI homolog	M.PabI homolog
26695	NC_000915	NP_207301/NP_207302	NP_207300
J99	NC_000921	NP_223173	NP_223172
HPAG1	NC_008086	YP_627220	YP_627219
Shi470	NC_010698	Replaced by *hrgC*	
G27	NC_011333	YP_002266092	YP_002266091
P12	NC_011498	YP_002301145	YP_002301144
B38	NC_012973	WP_000052859/WP_001048383	WP_025446089
B8	NC_014256	YP_003728709/YP_003728710	YP_003728708
PeCan4	NC_014555	Replaced by *hrgC*	
SJM180	NC_014560	YP_003928417	YP_003928416
ELS37	NC_017063	WP_001290273/WP_000052862	WP_000052863
52	NC_017354	Replaced by *hrgC*	
V225d	NC_017355	Replaced by *hrgC*	
908	NC_017357	YP_005765004	YP_005765003
Cuz20	NC_017358	Replaced by *hrgC*	
Sat464	NC_017359	Replaced by *hrgC*	
35A	NC_017360	WP_041201279	WP_041201231
SouthAfrica7	NC_017361	WP_014534762	Disrupted
Lithuania75	NC_017362	Disrupted	Disrupted
F30	NC_017365	Replaced by *hrgC*	
F32	NC_017366	YP_005775709	YP_005775708
F57	NC_017367	YP_005777249	YP_005777248
F16	NC_017368	YP_005779085	YP_005779086
Gambia94/24	NC_017371	YP_005780238	YP_005780237
India7	NC_017372	YP_005782183	YP_005782184
2017	NC_017374	YP_005783469	YP_005783468
83	NC_017375	Replaced by *hrgC*	

SNT49	NC_017376	WP_001048401	Disrupted
Puno120	NC_017378	Replaced by *hrgC*	
Puno135	NC_017379	Replaced by *hrgC*	
2018	NC_017381	YP_005791311	YP_005791310
51	NC_017382	YP_005793205	YP_005793206
HUP-B14	NC_017733	YP_006219552	YP_006219553
Shi417	NC_017739	Replaced by *hrgC*	
Shi169	NC_017740	Replaced by *hrgC*	
Shi112	NC_017741	Replaced by *hrgC*	
PeCan18	NC_017742	WP_029657379	WP_014662344
XZ274	NC_017926	YP_006338505	YP_006338506
Rif1	NC_018937	YP_006892989/YP_006892990	Disrupted
Rif2	NC_018938	YP_006936030/YP_006936031	Disrupted
Aklavik117	NC_019560	YP_007017054	YP_007017053
Aklavik86	NC_019563	YP_007018898/AFX89836	YP_007018897
OK113	NC_020508	YP_007536601	YP_007536600
OK310	NC_020509	Replaced by *hrgC*	
UM032	NC_021215	YP_007979503	YP_007979502
UM299	NC_021216	YP_007980042	YP_007980041
UM037	NC_021217	YP_007982157	YP_007982156
UM066	NC_021218	YP_008357885	YP_007983607
UM298	NC_021882	YP_008337421	YP_008337420
SouthAfrica20	NC_022130	YP_008471497/YP_008471498/YP_008471499	YP_008471496
BM012A	NC_022886	YP_008827304/YP_008827305	YP_008827303
BM012S	NC_022911	YP_008846633/YP_008846634	YP_008846632
Sheeba	NC_008229	YP_664607/YP_664608	YP_664606
MIT 99-5656	NC_017735	YP_006220545	YP_006220546

Sheeba, *H. acinonychis*; MIT 99-5656, *H. cetorum*. All other strains are *H. pylori*. Strains belonging to hspEAsia are colored in blue and those belonging to hspAmerind in red. In cases where more than two genome sequences are reported for one strain, only one genome is shown.
