# Supplementary material for: Transmission of the PabI family of restriction DNA glycosylase genes: mobility and long-term inheritance
Source: BMC Genomics. 2015 Oct 19;16:817. doi: 10.1186/s12864-015-2021-3 (PMC4615327; doi:10.1186/s12864-015-2021-3)
Supplement: Additional file 3: Figure S1. — Intergenic sequences between orthologous M.PabI homologs and upstream genes. Start codons for the M.PabI homologs are in bold and stop codons for upstream genes are shaded. Under the nucleotide sequences, encoded protein sequences are shown. (PDF 76 kb) [file 12864_2015_2021_MOESM3_ESM.pdf]

Figure S1. Intergenic sequences between orthologous M.PabI homolog genes and upstream genes. Start codons for M.PabI homolog are in bold and stop codons for upstream genes are shaded. Under the nucleotide sequences, encoded protein sequences are shown.

(A) H-1 (*H.* sp. MIT 01-6451, 03-1614 and *H. typhlonius*)

```
01-6451     GCGAAAAGCTATAAAGAACTTTGCGCTTTGTGAATCTGAAGCTTGAAAAAATATCTATTAG--------TAGCAA
             A  K  S  Y  K  E  L  C  A  L  *
03-1614     GCAAAAAGTTATAAAGAACTTTGTGCTTTGTAAATCTGAAGCTT-AAAAATATCTATTAGCATATTTATTGCAA
             A  K  S  Y  K  E  L  C  A  L  *
typhlonius  GCAAAAAGTTATAAAGAACTTTGTGCTTTGTAAATCTGAAGCTT-AAAAATATCTATTAGCATATTTATTGCAA
             A  K  S  Y  K  E  L  C  A  L  *


01-6451     --------CATTGAGTGTAAAGTAAGAATAGAATTAAATGACAGCTCAAGCACATTTAGACAGAATCCAA
                                                 M  T  A  Q  A  H  L  D  R  I  Q
03-1614     TATTCAACCATTGAGAGTAAAGCGAGAACAGAA-TAAATGACAGCCCAAGCACATTTAGATAGAATCCAA
                                                 M  T  A  Q  A  H  L  D  R  I  Q
typhlonius  TATTCAACCATTGAGAGTAAAGCGAGAACAGAA-TAAATGACAGCCCAAGCACATTTAGATAGAATCCAA
                                                 M  T  A  Q  A  H  L  D  R  I  Q
```

(B) H-2 (*H. muridarum* ST1) & C-4 (*C. concisus* UNSW2)

```
ST1         GATTCTATCTTTAAGCGGGTGTTTGGGGAGGGGAGATGAATAAAAAAACATTTAGTTTTGGAATC//
             D  S  I  F  K  R  V  F  G  E  G  R  *
ST1       //AGACAATGTTTTAAGAGGGTTTTTAGATAA--ATGACAGCACAAACACACCTAAGCACCAAGGAA
                                            M  T  A  Q  T  H  L  S  T  K  E
UNSW2       GATAAAATTTTTAAGAGAGTATTTGAAAAATAATGTCGATACAAAGACACTTAAATAAAATCGAT
             D  K  I  F  K  R  V  F  E  K  *
                                            M  S  I  Q  R  H  L  N  K  I  D
```

(C) H-3 (*H. bilis* ATCC 43879, *H. cinaedi* CCUG 18818, ATCC BAA-849, PAGU611, *C. upsaliensis* RM3195) & C-1 (*C.* sp. MIT 97-5078)

```
ATCC 43879  TCAAAACCCCCGGGAACTATTGAGTGGGAGTAGAGATAATTCAATGACAAAACCAACGCATTTAAGCACACAAGAA
             S  K  P  P  G  T  I  E  W  E  *           M  T  K  P  T  H  L  S  T  Q  E
CCUG 18818  TCAAAACCCCCGGGAACGATTGAGTGGGAGTAGAGATAATTCAATGACAAAACCAACGCATTTAAGCACACAAGAA
             S  K  P  P  G  T  I  E  W  E  *           M  T  K  P  T  H  L  S  T  Q  E
BAA-847     TCAAAACCCCCGGGAACGATTGAGTGGGAGTAGAGATAATTCAATGACAAAACCAACGCATTTAAGCACACAAGAA
             S  K  P  P  G  T  I  E  W  E  *           M  T  K  P  T  H  L  S  T  Q  E
PAGU611     TCAAAACCCCCGGGAACGATTGAGTGGGAGTAGAGATAATTCAATGACAAAACCAACGCATTTAAGCACACAAGAA
             S  K  P  P  G  T  I  E  W  E  *           M  T  K  P  T  H  L  S  T  Q  E
RM3195      TCAAAACCCCCGGGAACGATTGAGTGGGAGTAGAGATAATTCAATGACAAAACCAACGCATTTAAGCACACAAGAA
             S  K  P  P  G  T  I  E  W  E  *           M  T  K  P  T  H  L  S  T  Q  E
MIT 97-5078 AGCAAACCCCCAGGAACCATTGAGTGGGAGTAGGGA-------ATGACGATACAAGCACATTTAAATAAAATCCAA
             S  K  P  P  G  T  I  E  W  E  *           M  T  I  Q  A  H  L  N  K  I  Q
```

(D) H-4 (*H. suis* HS1 and *H. heilmannii* ASB1.4)

```
HS1         CCTTTGAAGTGATTTTACAGACTATGCAAGAGGTGTTGTTATAAAACCTTCACATCTGCATTACTCCGAGCAAATCAATTT
            P  F  E  V  I  L  Q  T  M  Q  E  V  L  L  *
                                                     M  K  P  S  H  L  H  Y  S  E  Q  I  N  L
HS1         CCTTTGAAGTGATTTTACAGACTATGCAAGAGGTGTTGTTATAAAACCTTCACATCTGCATTACTCCGAGCAAATCAATTT
            P  F  E  V  I  L  Q  T  M  Q  E  V  L  L  *
                                                     M  K  P  S  H  L  H  Y  S  E  Q  I  N  L
ASB1.4      ACAGCTCCTTAGTGCAAGCAACCATGCAAGAAGTGCTAAATTGA------GCCACCTAAGCCCCCTTGAAAAGATCAATCT
            N  S  S  L  V  Q  A  T  M  Q  E  V  L  N  *
                                                     M        S  H  L  S  P  L  E  K  I  N  L
```

(E) H-9 (*H. pylori* F16, J99, *H. acinonychis* Sheeba, *H. cetorum* 00-7128 and 99-5656)

```
F16         CATGCTAAAGATGTGAAGCAGCTAGATGTGTAAGGGATTTTGATTA---ACCCTAAACATTTAAATAAGCGAGAG
             H  A  K  D  V  K  Q  L  D  V  *        M  I     N  P  K  H  L  N  K  R  E
J99         CATGCCAAAGATGTGAAGCAACTAGATGTGTAAGGGGTTTTGATTAGAACCCCCAAGCATTTAACCAAGCAAGAG
             H  A  K  D  V  K  Q  L  D  V  *        M  I  R  T  P  K  H  L  T  K  Q  E
Sheeba      CATGCTAAAGATGTGAAACAATTGGATGTGTAAGGGATTTTAATCACTACCCCTAAACATTTAACCAAGCAAGAG
             H  A  K  D  V  K  Q  L  D  V  *           M  T  T  P  K  H  L  T  K  Q  E
00-7128     CATGCTAAAGATGTGAAACAACTAGATGTTTAAGGAAAATTTATCACAACCCCTAAACATTTAACCAAACAAGAG
             H  A  K  D  V  K  Q  L  D  V  *           M  T  T  P  K  H  L  T  K  Q  E
99-5656     CATGCTAAAGATGTGAAACAACTAGATGTGTGAATAATGTTTTGTACCGGCAATTTTAGTTCGGTATGGCTATCT//
             H  A  K  D  V  K  Q  L  D  V  *
            CCCTAGAGTGAGCTATTTTTTATTATTACTAAGGAGTTTTGTATCACAACCCCTAAACATTTAACTAAACAAGAA
                                                       M  T  T  P  K  H  L  T  K  Q  E
```

(F) C-3 (*C. jejuni* ATCC 33560, *coli* LMG 23336, *coli* H56)

```
33560       ATCAAGAGTTTGCAAGAATATTTAAAGGACTAGTATTGATGCAAAACCATCTTAACAGAGAAATAATGATT
             I  K  S  L  Q  E  Y  L  K  D  *     M  Q  N  H  L  N  R  E  I  M  I
LMG23336    ATCAAGAGTTTGCAAGAATATTTAAAGGACTAGTATTGATGCAAAACCATCTTAACAGAGAAATAATGATT
             I  K  S  L  Q  E  Y  L  K  D  *     M  Q  N  H  L  N  R  E  I  M  I
H56         ATCAAGAGTTTGCAAGAATATTTAAAGGACTAGTATTGATGCAAAACCATCTTAACAGAGAAATAATGATT
             I  K  S  L  Q  E  Y  L  K  D  *     M  Q  N  H  L  N  R  E  I  M  I
```
