# Supplementary material for: Transmission of the PabI family of restriction DNA glycosylase genes: mobility and long-term inheritance
Source: BMC Genomics. 2015 Oct 19;16:817. doi: 10.1186/s12864-015-2021-3 (PMC4615327; doi:10.1186/s12864-015-2021-3)

Figure S2. Accumulation of R and M genes around the PabI family of RM system in *H. muridarum*. The arrows represent genes. Gene numbers are shown above the arrows. The R and M gene pairs are indicated by brackets. Target sequences are presented below the RM pairs or M genes. Gene 3185 is a partial duplication of gene 3170. The sequences of genes 3210 and 3175 showed no significant sequence similarities.

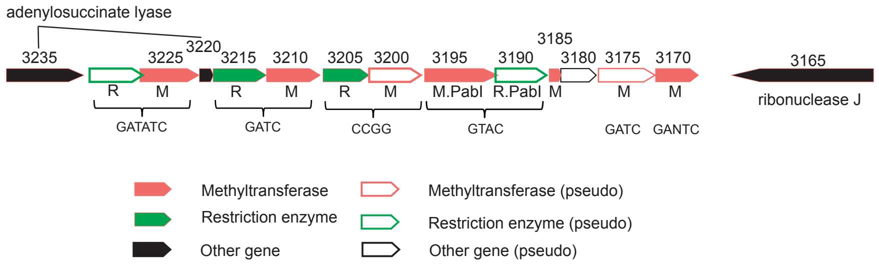

Supplement: Additional file 4: Figure S2. — Accumulation of R and M genes around the PabI family of RM system in H. muridarum. The arrows represent genes. Gene numbers are shown above the arrows. The R and M gene pairs are indicated by brackets. Target sequences are presented below the RM pairs or M genes. Gene 3185 is a partial duplication of gene 3170. The sequences of genes 3210 and 3175 showed no significant sequence similarities. (PDF 878 kb) [file 12864_2015_2021_MOESM4_ESM.pdf]
