# Supplementary material for: Transmission of the PabI family of restriction DNA glycosylase genes: mobility and long-term inheritance
Source: BMC Genomics. 2015 Oct 19;16:817. doi: 10.1186/s12864-015-2021-3 (PMC4615327; doi:10.1186/s12864-015-2021-3)
Supplement: Additional file 5: Figure S3. — Sequence comparisons of M.PabI and R.PabI homolog fragments. Sequences of H. acinonychis Sheeba and H. pylori G27 are in the “mcrB” locus, while other sequences are in the “pabI” locus. The sequences that do not correspond to M.PabI or R.PabI homologs are colored in either red or blue. Nucleotides distinct from the M.PabI or R.PabI homolog sequence are shaded in the alignable region. The start codon (TTG) for M.PabI homolog and stop codon (TAA) for R.PabI homolog are in bold. (PDF 56 kb) [file 12864_2015_2021_MOESM5_ESM.pdf]

Figure S3. Sequence comparison of M.PabI and R.PabI homolog fragments. The sequences of *H. acinonychis* Sheeba and *H. pylori* G27 are at the "mcrB" locus, while other sequences are at the "pabI" locus. The sequences not corresponding to M.PabI or R.PabI homolog sequences are shown in either red or blue. The nucleotides not identical to the M.PabI or R.PabI homolog sequence are shaded in the alignable region. The start codon for M.PabI homolog and the stop codon for R.PabI homolog are in bold.

M.PabI homolog

J99	**TTG**ATTAGAACCCCCAAGCATTTAACCAAGCAAGAGAGCGTGAATTTAGGGGCTTACTACACGCCCCCTT
Sheeba	TTGATTAGAACCCCTAAACATTTAACCAAGCAAGAGAGCGTGAATTTAAGGGCTTACTACACGGCCCCTT
G27	TTGATTAGAACCACTAAACATTTAACCAAACAAGAGAG--TGGATTTAGGAGCTTACTGCACGCCACCTT
HpA-26	TTGATTAGAACCCCTAAACATTTAACCAAGCAAGAGAGCGTGAATTTAGGGGCTTACTACACGCCTTGTT
oki102	TTAATCGCCACTCCTAAACATTTAAATCAGCGAGAGAGCGTGAATTTAGGGGCTTATTACACGCCTTGTT
F30	TTGATTAGAACCCCTAAACATTTAAATAAGCGAGAGAGCGTGAATTTAGGGGCTTATTACACGCCCTGTT
Puno120	TTGATTAGAACCCCTAAACATTTAAATAAGCGAGAGAGCGTGAATTTAGGGGTTTATTACACGCCTTGTT

J99	ATTTAGTGGATTGCGCTTACAAGCTTTTAAAAAAGCATGTTGGTATTGAAAACTACACGCTTTTAGACAC
Sheeba	ATTTAGTGGGTTGTGATTGCAAGCTTTTAAAAAGGCATGTTAATATTGAAAAACACACACTTTTATACAC
G27	ATTTAGTGGATTATGCTTACAAACTTTTAAAAAAGCATGTTGGTATTGAAAAATACACGCTTTTAGACAC
HpA-26	ATTGAGTGCAGG-AAACCGCTAGGCGATCATACCCCTTGATCGCAAGATACCCCTAGAGTATTCATCGGT
oki102	ATTGAGTGCAGG-AAACCGCTAGGCGATCATACCCCTTGATCGCAAGATACCCCTAGAGTATTCATCGGT
F30	ATTGAGCGTGGGGAAAGCACTAGGCGATTATACCTTTTGATCGCAAGATACCCCTAGAGTATTCATCAGT
Puno120	ATTGAGTGTGAGA-AAGCGCTAGGCGATCATACCTTTTGATCGCAAGATACCTCTAGAGTATTCATCAGT

J99	CGCATGTGGTAATAAAGAGTTTTTAAAGCTCCACCACCCTAAAAAAATAGGAGCGGATATTGACCCTAAG
Sheeba	CGCTTATGGCAATAAAGAGTTTTGAACTGATTATATGGCAAAATTTTCCTCAAGGAATAATGCCTTTTGC
G27	CGCTTAAACGCAACAAAACTTTTTGA--------------------TTTCTAATAAATAATGCCTTTTGC
HpA-26	GGGTTCGTAACCGCTTTCTAAAAGTTTGGTTGTGTATTGCTTGTTGTAGATAAAGGCAAATACTATATTG
oki102	GGGTTCGTAACCGCTTTCTAAAAGTTTGGTTGTGTATTGCTTGTTGTAGATAAATGCAAATACTATATTG
F30	GGGTTCATAACCGCTTTCTAAAAGTCTGGTCGTGTATTGTTTGTTGTAGGTAAATGCAAATATTGTATTA
Puno120	GGGTTCATAGCCGCTCTCTAAAAGTTTGGTTGTGTATTGTTTGTTATAGATAAAGGCAAATATAATTATA

R.PabI homolog

J99	GCCATGCTGTATTTTTGCTTTTCTATTTTGGAGTTAAAAACCGCTACCCCCTTATTAAACAGAACCGCTA
HpA-26	GAGAGAAGTCAAGGGGTTTGATCAAAAAAAGGCTTTATTTTGATTGAATAAAATTATCGCTTGCTCACAC
oki102	GAGAGAAGTCAAGAGGTTTAATCAAAAAAGGCTT--ATTTTGATTGAATAAAATTATCGCTTGTCCACAC
F30	GAGAGAAGTCAAGGGGTTTGATCAAAAAAGGCTTT-ATTTTGATTGAATAAAATTATCGCTTGCTCACAC
Puno120	GAGAGAAGTCAAGGGGTTTGATCAAAAAAGGCTTT-ATTTTGATTGAATGAAATTATCGCTTGCTCACAC

J99	TGCCCAAAGAACATGCCCTTTTGATTATCCATGAAACCAACGCTCTTGTGTTTTTAGAAATGCTTAAAAT
HpA-26	AAGCTTTTTGGTTATTGGATTGTAAAAAA-TACTAAAAAGCATTTTTTAACCTTTAGAAATGCTTAAAAT
oki102	AAGCTCTTTGATTATTGGATTGTAAAAAA-TACTAAAAAGCATTTTTTAACTTTTAGAAATGCTTAAAAT
F30	AAGCTCTTTGGTTATTGGATCGTAAAAAA-TACTAAAAAGCATTTTTTAACCTTTAGAAATGCTTAAAAT
Puno120	AAGCTCTTTGGTTATTGGATTATAAAAAAATACTAAAAAGCATTTTTTAACCTGTAGAAACGCTTAAAAT

J99	TTTTGGACTTTTAAGCCAAGTGCACCATAACGATGTGTTAAAGATTTTAGAAAAAATACTTCAAAAT**TAA**
HpA-26	TTTTGGACTTTTAAGCCAAGCGCACCATAGCGATGTGTTAAAGATTTTAGAAAAAATACTTCAAAATTAA
oki102	TTTTGGACTTTTAAGCCAAGCACACCATGACGATGTGTTAAAGATTTTAGAAAAGATACTTCAAAATTAA
F30	TTTTGGACTTTTAAGCCAAGCGCACCATGACGATGTGTTAAAGATTTTAGAAAAAATACTTCAAAATTAA
Puno120	TTTTGGACTTTTAAGCCAAGCGCACCATGACGATGCGTTAAAGATTTTAGAAAAGATACTTCAAAATTAA
